# Determining epitope specificity of T-cell receptors with transformers

**DOI:** 10.1093/bioinformatics/btad632

**Published:** 2023-10-17

**Authors:** Abdul Rehman Khan, Marcel J T Reinders, Indu Khatri

**Affiliations:** Department of Intelligent Systems, Delft University of Technology, Delft 2600 GA, The Netherlands; Department of Intelligent Systems, Delft University of Technology, Delft 2600 GA, The Netherlands; Leiden Computational Biology Center, Department of Molecular Epidemiology, Leiden University Medical Center, Leiden 2333 ZA, The Netherlands; Leiden Computational Biology Center, Department of Molecular Epidemiology, Leiden University Medical Center, Leiden 2333 ZA, The Netherlands; Department of Immunology, Leiden University Medical Center, Leiden 2333 ZA, The Netherlands

## Abstract

**Summary:**

T-cell receptors (TCRs) on T cells recognize and bind to epitopes presented by the major histocompatibility complex in case of an infection or cancer. However, the high diversity of TCRs, as well as their unique and complex binding mechanisms underlying epitope recognition, make it difficult to predict the binding between TCRs and epitopes. Here, we present the utility of transformers, a deep learning strategy that incorporates an attention mechanism that learns the informative features, and show that these models pre-trained on a large set of protein sequences outperform current strategies. We compared three pre-trained auto-encoder transformer models (ProtBERT, ProtAlbert, and ProtElectra) and one pre-trained auto-regressive transformer model (ProtXLNet) to predict the binding specificity of TCRs to 25 epitopes from the VDJdb database (human and murine). Two additional modifications were performed to incorporate gene usage of the TCRs in the four transformer models. Of all 12 transformer implementations (four models with three different modifications), a modified version of the ProtXLNet model could predict TCR–epitope pairs with the highest accuracy (weighted F1 score 0.55 simultaneously considering all 25 epitopes). The modification included additional features representing the gene names for the TCRs. We also showed that the basic implementation of transformers outperformed the previously available methods, i.e. TCRGP, TCRdist, and DeepTCR, developed for the same biological problem, especially for the hard-to-classify labels. We show that the proficiency of transformers in attention learning can be made operational in a complex biological setting like TCR binding prediction. Further ingenuity in utilizing the full potential of transformers, either through attention head visualization or introducing additional features, can extend T-cell research avenues.

**Availability and implementation:**

Data and code are available on https://github.com/InduKhatri/tcrformer.

## 1 Introduction

The human immune system can mount the immune response by generating multiple T-cell receptors (TCRs) in response to a pathogenic infection. Principally, this response involves interaction between TCRs (antigen/epitope-recognition receptors on T cells) and the epitopes (short peptides from pathogenic proteins) present in infectious agents (bacteria/viruses). Complementarity determining region 3 (CDR3) on both the α and β chains of TCRs binds with the epitope. The diversity of TCRs is estimated to be ∼10^18^ in humans and 10^15^ in mice. The hypervariability of the CDR3 region is imparted during the *V(D)J* recombination process followed by the junctional diversity due to the insertion of additional bases during the recombination process (see Supplementary Text for more details). Determining the binding specificity of such highly diverse and variable TCR sequences to an epitope is a challenging problem since multiple TCRs can bind to an epitope and similar antigenic peptides can be recognized by a multitude of TCRs. Learning the specificity of TCRs to the epitopes will enhance our understanding of the specificity of the immune responses at the receptor level to multiple similar epitopes and/or different epitopes from different infectious agents.

An alignment model or attention focuses a neural network on learning relevant relationships, reducing heavy translations and improving translation performance. One of the deep neural architectures using attention is transformers (Vaswani *et al.* 2017). Unlike long short-term memory models, transformers are not restricted by the length of input sequences. Transformers are trained using transfer learning, in which transformer models are first pre-trained on task-analogous objectives and later fine-tuned on task-oriented objectives (see Supplementary Text for more details). Through transfer learning, a transformer can learn contextual information from a large dataset during pre-training and then apply the knowledge learnt from pre-training towards a downstream task, which can be applied to small datasets. Since the introduction of transformers, many variations of its architecture have been released (Lan *et al.* 2020, Yang *et al.* 2019, Clark *et al.* 2020, Elnaggar *et al.* 2020). Common in all these transformer architectures is the self-attention mechanism. While attention focuses on important parts of the input that lead to a better conclusion (e.g. classification), self-attention focuses on surrounding words to delineate the meaning between similar words. Furthermore, multi-headed self-attention learns multiple representations from the same input; each of these are termed as a head (Devlin *et al.* 2018). Different transformer models implement different strategies to utilize attention mechanisms efficiently.

Transformers are not just limited to natural languages; given a sufficiently large corpus, they can be tailored towards any sequence-based inference. ProtTrans is a transformer model trained on a large corpus of protein data, i.e. the Uniref database and the Big Fantastic Database (BFD), comprising 216 million and 2.122 billion protein sequences, respectively, and has been shown to learn the contextual grammar of amino-acid sequences (Elnaggar *et al.* 2020). Analogous to Natural Language processing (NLP) tasks, the ProtTrans pre-training task involved a vocabulary of 20 amino acids. Therefore, sentences and words are analogous to protein sequences and amino acids, respectively. Four variants of this ProtTrans transformer model, three auto-encoders [ProtBERT (Devlin *et al.* 2018), ProtAlbert (Lan *et al.* 2020), ProtElectra (Clark *et al.* 2020)] and an auto-regressive model [ProtXLNet (Yang *et al.* 2019)], are publicly available as of November 2021. The attention mechanisms of these models find relevant regions in a protein sequence (pattern of amino acids) that accurately emulate the knowledge needed to understand the mechanisms behind a protein function. This inspired us to use transformers to learn the patterns of a TCR–epitope interaction to understand the processes determining the affinity between a TCR and an epitope.

This work demonstrates the feasibility of transformers to predict the TCR specificity to individual epitopes based on TCR sequence information. We adapted the pre-trained transformers to predict TCR–epitope binding specificity and compared performances not only between different transformer models (ProtBERT, ProtAlbert, ProtElectra, and ProtXLNet) but also compared outcomes of these transformer models to previously available tools based on distance metrics (Jokinen *et al.* 2021) or deep learning (Sidhom *et al.* 2021). Transformers are known to accept only sequence data as input; however, it is known that additional information about a TCR (besides their sequence), such as gene name and major histocompatibility complex (MHC) class, can enhance the predictive power of the models (Jokinen *et al.* 2021, Sidhom *et al.* 2021). In comparison to other existing tools, we uniquely modified the publicly available transformers to incorporate information about TCR gene names, as *V* and *J* genes are instrumental in providing specificity to the TCRs to recognize a epitope (Hodges *et al.* 2003). Altogether, we showed that transformers can be used to predict TCR binding specificity to unique epitope.

## 2 Materials and methods

### 2.1 Data acquisition and preparation

VDJdb (Shugay *et al.* 2018) provides a centralized source for TCR–epitope pairs. For a TCR, the database provides CDR3 sequence, and *V* and *J* gene names of the TCRβ and/or TCRα receptor protein, the MHC Class I/II annotation, as well as the organism (e.g. human, mouse) in which it was observed (Shugay *et al.* 2018). For epitopes, the database provides the epitope sequence (e.g. TVYGFCLL), the parent gene of the epitope (e.g. m139), and the antigen species for the epitope (e.g. MCMV). Confidence scores (0, 1, 2, and 3) are associated with each TCR–epitope pair, wherein a zero score indicates computationally predicted specificities, and higher scores (i.e. 1, 2, and 3) represent pairs that were validated using one or more wet-lab-based techniques, e.g. assay identification, TCR sequencing, or verification procedure.

Data obtained from VDJdb consisted of 81,762 entries (as per November 2021), which included epitopes binding to a variety of antigens from cancer, immune-disorders, plants, microbes, etc. We filtered the dataset by selecting TCR-β chains of Human and Murine epitopes with a confidence score >0. Only single instances of duplicate CDR3 sequences with the same *V* gene, *J* gene, MHC A, and MHC B were retained; however, MHC gene was not used to improve performance in our study. Additionally, we removed epitope species not originating from infectious agents, i.e. related to autoimmune disorders, cancer, synthetic, bacterial antigens, or allergies. Finally, the TCR–epitope pairs (called ‘classes’ from hereinafter) comprising of minimum 50 CDR3 sequences were retained. Table 1 summarizes the number of TCR–epitope pairs left after different filter steps. Finally, 2674 pairs have been used for training, validating, and testing purposes. This consisted of 25 unique TCR–epitope pairs (classes); 10 classes with more than 100 instances (these were considered ‘Easy-to-classify’) and 15 classes with <100 instances (these were considered ‘Hard-to-classify’) (Supplementary Fig. S1). Information about unique *V* and *J* genes (63 *V* genes and 13 *J* genes) was encoded using an ordinal encoder and associated with each TCR sequence. Consequently, the class label for each TCR–epitope pair consists of the Epitope’s species, genes, and sequence, e.g. ‘MCMV m139 TVYGFCLL’ class label is composed of MCMV (Epitope’s species), m139 (Epitope’s gene), and TVYGFCLL (Epitope’s sequence).

**Table 1.** Number of TCR–epitope pairs retrieved from the VDJdb database after each filtering step.

Filtration criteria	Sample count
Raw data	81 762
Post removing N/A	80 679
Selecting only human and murine antigen	78 647
Selecting TCR–epitope pairs with confidence score >0	9580
Post removing allergen and cancer antigen	8547
Post removing duplicated entries^a^	5680
Selecting the TCR–epitope pairs with TRB sequences	4057
Selecting the TCR–epitope pairs with ≥50 instances	2674

a Duplicates were removed based on the same gene and MHC gene name and identical CDR3 sequence.

### 2.2 Multi-class classification of TCR–epitope pairs

Four different models, i.e. ProtBERT (Devlin *et al.* 2018), ProtAlbert (Lan *et al.* 2020), ProtElectra (Clark *et al.* 2020), and ProtXLNet (Yang *et al.* 2019) were used for learning and predicting the TCR–epitope pairs in a multi-class setting (Elnaggar *et al.* 2020). The input is TCR protein sequence, and the output is 1 of 25 epitope classes. We used pre-trained models where ProtBERT, ProtAlbert, and ProtXLNet models were pre-trained on the Uniref database (Suzek *et al.* 2007), and ProtElectra was trained on the BFD database (https://bfd.mmseqs.com/). These pre-trained models were subsequently used to learn and predict TCR–epitope specificity. Hereto, the 2,674 TCRs divided over 25 different classes of epitopes is split into 70% training, 15% validation, and 15% testing dataset in a stratified way.

#### 2.2.1 *Using pre-trained transformers to predict TCR–epitope specificity*

**Figure 1.**
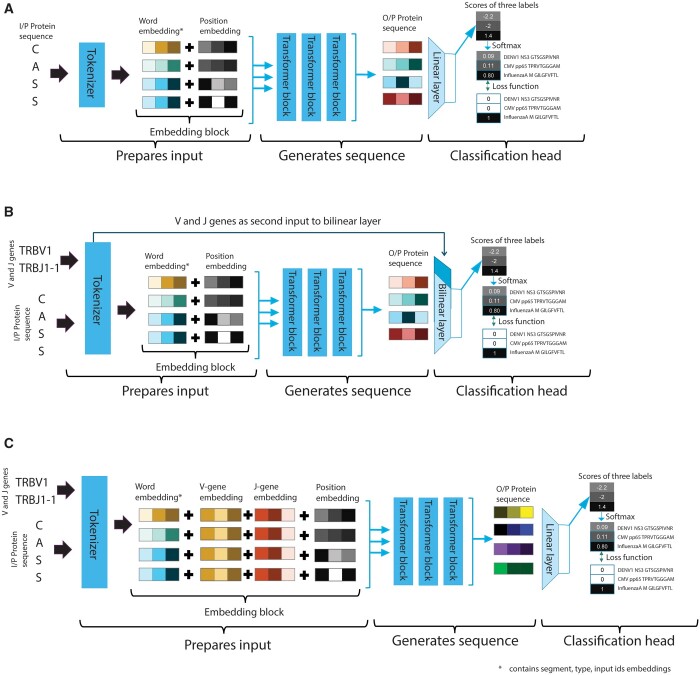
Overview of the data flow in the different transformers settings. (A) *Baseline setting*: The CDR3 sequence is provided to the tokenizer to generate a numerical representation of the input sequence based on embeddings learnt during pre-training. The prepared input is divided into three parts (Query, Value, and Key) and provided to the transformer block to implement the self-attention mechanism. The output representation generated by the transformer block is then used by the classification block to predict the epitope label. (B) *Classification setting*: Sequence is provided to the transformer block and the gene usage information is directly presented to the classification head, where the gene information is added into a bilinear layer. (C) *Embedding setting*: The sequence along with the gene usage information from the embedding block is provided to the transformer block; thereafter, the combined embeddings are fine-tuned.

Each transformer has its specific tokenizer, which prepares the sequence-based input using its pre-trained vocabulary. A general overview of the transformer model is depicted in Fig. 1A. The embedding block prepares input (CDR3 sequences of TCRB in selected TCRs) for the transformer, which includes adding special tokens to denote special relationships (such as padding), an attention mask to denote if a token is to be considered for attention calculation, and a segment ID to denote two separate sequences in the same input. The tokenizer in the embedding block also performs the encoding of labels and presents sequences to the transformer block. Each tokenizer maintains homogeneity of the sequence encoding and presents each sample to the transformer to learn a representation. The representation of the input sequence is then fed to a classification block, which performs sequence inference tasks. In the baseline transformer (Fig. 1A), the classification block receives an *n*-dimensional (e.g. in the case of ProtBERT, *n* = 768) representation of each amino acid as input from the final hidden state of the transformer block. The first token of every sequence is a special classification token (signified as Classification (CLS) by the tokenizer), which contains the aggregated representation of the input sequence in the final hidden state. This representation is termed pooled output. The pooled output is the input to the classification block to perform the TCR–epitope classification task. The standard classification block consists of a linear layer mapping the pooled output vector (x of the size of the hidden layer) from the transformer block as shown in Equation (1) to class label (y), where y is the number corresponding to the classes (epitope labels e.g. ‘MCMV m139 TVYGFCLL’).


(1) $$y=\;xA^{T}+b.$$

#### 2.2.2 *Loss function for imbalance data*

The imbalance in our dataset prompted us to adapt the loss function. A binary loss function created upon cross-entropy is Focal-loss (Lin *et al.* 2020) which adds a modulating factor (or Focusing parameter) to cross-entropy [Equation (2)]. The modulating factor is adjusted using gamma (γ >0), which reduces the contribution of samples from the ‘easy-to-classify’ classes and enhances the contribution of samples of the ‘hard-to-classify’ classes to the loss value. The enhanced loss value of samples from the hard-to-classify classes informs the model to accommodate more for these classes, which otherwise is overlooked during training. The loss appears normal to the model; the adjusted loss value is the manipulation performed before providing it to the model, and the model can then direct the gradient accordingly.


(2) $$\mathrm{CE}\;=\;-\sum_{i=0}^{c}t_{i}l\mathrm{og}(f{\left(s\right)}_{i}).$$

(3) $$\mathrm{FL}\;=\;-\sum_{i=0}^{c}t_{i}{\left(1-{f\left(s\right)}_{i}\right)}^{\gamma }l\mathrm{og}(f{\left(s\right)}_{i}).$$

Multi-class cross-entropy can be extended to multi-class focal loss with an additional step after calculating the sum of losses for each class, i.e. applying the modulating factor as shown in Equation (3). A recommendation of γ=2 was made by the authors of focal loss; however, they only assumed binary cases. For the multiclass classification model in our problem, we used γ as hyperparameter to optimize.

#### 2.2.3 *Providing VJ gene (non-sequence) information to the transformers*

We sought ways to include information about gene names (e.g. *TRBV1* and *TRBJ1-1*) as additional input as this has shown to improve TCR–epitope binding specificity prediction performance. To avoid re-training the transformer models (which would take an enormous amount of time, see Supplementary information: Transfer learning), we altered the tokenizer to accommodate additional features along with sequences. We explored two different ways to do that: (i) before, and (ii) after the transformer blocks. The first option requires a modification in the classification block of the model (Fig. 1B). The classification block receives the additional features directly from the tokenizer, bypassing the transformer block. Each CDR3 sequence has *V* and *J* gene names associated with it. An ordinal encoding for these gene names is provided to the tokenizer, which it associates with each sample and then given to the classification block. We replaced the linear layer with a bi-linear layer where the pooled output ($x_{1}$ having a size equal to the size of the hidden layer) and gene encoding ($x_{2}$ with size 2 representing *V* and *J* gene names separately) is mapped to a class label (y). The bi-linear transformation, as shown in Equation (4), will calculate weight matrix (*A*) and bias (*b*) based on the two input vectors (sequence, *x*_1_, and gene name information, *x*_2_), which will express the interaction between sequence and gene names.


(4) $$y=\;x_{1}^{T}Ax_{2}+b.$$

The second option requires a modification in the embedding block (Fig. 1C). The embedding block of a pre-trained model contains a learnt representation of its vocabulary. The introduction of additional information would require learning new representations. To avoid this, we propose to add two embedding layers; one for all unique *V* genes, and another for all unique *J* genes (Fig. 1C). The padding index for these layers is different from the padding for the word embedding layer (so we explicitly pass an alternative padding index for the additional features; 0 indices for *V* and *J* genes embedding layer). Special tokens were added before and after a protein sequence during tokenization. The separate padding index will associate a gene embedding to only a protein sequence and not to the special tokens. Each sequence embedding and the *V* and *J* gene names are then merged. The new representations were presented to the classification block.

The padding index, the unique gene names and the special token together, resulted in a total embedding size for *V* genes of 65, and for *J* genes of 15. These new layers were randomly initialized when fine-tuning. To synchronize with the fine-tuning of the rest of the model, these weights are learned at an increased rate (for the sake of simplicity, by a factor of 10).

### 2.3 Hyperparameter optimizations and performance evaluation

A Bayesian algorithm (Tree Parzen Estimator) is utilized to optimize the hyperparameters of different models using Optuna (Akiba *et al.* 2019), which is recommended for situations that require the exploration of many hyperparameters. Experiments are tracked using comet.ml and PyTorch for training each model. Optimization is done for 10 major hyperparameters: gradient accumulation, learning rate, weight decay, attention layer dropout (not included in auto-regressive or AR model), hidden layer dropout (‘dropout’ in AR model), classifier layer dropout (‘summary last dropout’ in AR model), adam_beta1, adam_beta2, warmup ratio, and gamma. A seed as a hyperparameter ensures model stability, but performance is not evaluated on seed values.

When optimizing hyperparameters we gain insight into the behaviour of the transformers when trained with TCR data. Optuna (Akiba *et al.* 2019) provides sampling strategies depending on the hyperparameter of choice. Supplementary Table S1 presents the sampling strategy for each hyperparameter and its range. Since training steps are directly dependent on training batch sizes, and since we have small training data of 1,871 samples, we used a training batch size and an evaluation batch size of 1 and 8, respectively. In preliminary experiments, we experienced high fluctuations in evaluation loss, which made transformer training susceptible to stopping prematurely. Therefore, each experiment is trained with an early stopping for 50 training epochs giving sufficient training time to adjust fluctuations caused by focal loss.

### 2.4 Evaluation metrics

We used the weighted F1-score and receiver operating characteristic (ROC) curve plots as metrics to assess the predictability of the transformer models. Additional metrics included were a balanced accuracy score, weighted precision, and weighted recall to provide an overview of the different performances of a model. Parallel coordinate plots and parameter importance were plotted to evaluate the contribution of parameters towards the objective value.

In a multi-class setting, the assessment of transformers is done through the epitope-specific perspective and model-specific perspective. The area under the ROC (AUROC) curve provides an epitope-specific perspective where performance comparison for TCR specificity can be made. The weighted F1-score of the model provides us with a holistic measure for comparing different models (across all methods) in a multi-class setting and hence a model-specific perspective.

The performances of all 12 transformer implementations (4 transformer models each with 3 different modifications) were evaluated using ROC plots. The AUROC curve for all 25 labels were assessed using the Wilcoxon test to compare performance across different implementations of the same transformer model. The transformer implementation with no modifications for each model are called baseline, while the ones with modifications in classification and embedding blocks are called classification and embedding.

We compared the transformer models to existing (non-transformer) models. TCRGP and TCRdist both have 24 common epitopes that overlap with our filtered dataset. DeepTCR has 20 epitopes in common. We used the AUC values published in these studies to compare with the AUC values of our models.

### 2.5 Benchmarking performance of our method

We benchmarked the performance of our methods by first assessing our models on the IMMREP benchmarking dataset (Meysman *et al.* 2023) and later by comparing the performance of our model with the model that is solely based on TCR sequence similarities (Baseline-SS) (Montemurro *et al.* 2021). Of 25 epitopes in our study, only four epitopes, i.e. CMV pp65 NLVPMVATV, CMV pp65 TPRVTGGGAM, EBV BMLF1 GLCTLVAML, and InfluenzaA M GILGFVFTL were common in the benchmarking dataset. Similarly, only four epitopes were common in the baseline-SS model as well, i.e. CMV pp65 NLVPMVATV, EBV BMLF1 GLCTLVAML, HCV NS3 ATDALMTGY, and InfluenzaA M GILGFVFTL. All these labels are hard-to-classify. For the Baseline-SS model, we compared the best performer for the four labels rather than our overall best performer. Finally, we assessed the performance of our best model is not by chance and can perform better than the *V* and *J* genes identities alone.

## 3 Results

We compared the performance of four different transformer models, three of which are based on an auto-encoder architecture (ProtBERT, ProtAlbert, and ProtElectra) and one is based on an auto-regressive structure (ProtXLNet), as well as their modification to include information about the *V/J* genes, on a dataset containing binding specificities between 2,674 TCR–epitope pairs. These pairs are split in 25 classes, with 10 classes having more than 100 instances (and considered ‘Easy-to-classify’), while 15 classes have <100 samples (and considered ‘Hard-to-classify’) (Supplementary Table S2). Data were split in 70% training, 15% validation, and 15% testing dataset in a stratified way across the classes. The validation set was used to optimize the hyperparameters, and the testing set was used to estimate the performance of the optimized models. Each of the four different transformer models were run in three different settings: (i) without the use of information about the *V/J* genes (Baseline setting), (ii) including the *V/J* gene names within the classification block (Classification setting), and (iii) including information about the *V/J* names by modifying the embedding layer (Embedding setting). See Fig. 1 and Section 2 for more details. This resulted in 12 optimized transformer models (4 different transformers, in 3 different settings). These optimized models were subsequently compared to three of the current best-performing tools classifying TCR–epitope pairs, i.e. TCRGP, TCRdist, and DeepTCR (all non-transformer-based). We first discuss the tuning of the hyperparameters and the learning behaviour of the transformer models. Then we compare the classification performances between the different models.

### 3.1 Learning behaviours of the transformer models

The three auto-encoder models had 11 hyperparameters (including the seed value), while the auto-regressive model had 10 hyperparameters). Optimization for these hyperparameters was performed for at least 100 runs for each implementation. For the ProtBERT and ProtElectra auto-encoder models, the hidden-layer dropout probability had the largest influence on the optimization (65%–87% and 71%–81%, respectively) (Table 2, Supplementary Fig. S2). Moreover, for both models, the importance of the hyperparameters is roughly the same across the three different settings. For the ProtAlbert auto-encoder, we observe equal influence across hyperparameters. Within the baseline setting, the hidden-layer dropout probability is still the most influential although the learning rate is also important, while in the other settings, the learning rate is the most influential (21–39%). This could be because ProtBert and ProtElectra do not have parameter sharing among the encoder blocks, resulting in a large network size (thus more parameters to train, emphasizing the need for regularization), whereas ProtAlbert have parameter sharing among the encoder blocks, resulting in a smaller network size and thus less need for regularization. As ProtAlbert is not solely dependent on the encoding representation but also on the learning rate, it might be more efficient in learning new relationships from the interaction between TCRs and epitopes.

**Table 2.** Performance metrics of the different transformer models across the three different settings (Baseline, Classification, and Embedding setting) on the test dataset.

PROT transformers	Settings	Duration (h)	Weighted F1-score	Balanced accuracy	Mean AUC
ProtBERT	Baseline	4.49	0.39	0.33	0.79
	Classification	1.99	0.41	0.37	0.8
	Embedding	2.21	0.41	0.35	0.79
ProtAlbert	Baseline	3.16	0.42	0.37	0.79
	Classification	0.57	0.38	0.33	0.78
	Embedding	3.21	0.46	0.44	0.81
ProtElectra	Baseline	1.29	0.46	0.41	0.86
	Classification	0.9	0.44	0.41	0.8
	Embedding	0.99	0.47	0.4	0.84
ProtXLNet	Baseline	1.57	0.48	0.44	0.81
	Classification	1.21	0.46	0.38	0.8
	Embedding	1.23	0.55	0.5	0.88

The overall importance of the hyperparameters in the Classification setting of the ProtXLNet auto-regressive model was similar to the ProtBert and ProtElectra auto-encoders, with the dropout probability being most influential (62%). The Baseline and Embedding settings for ProtXLNet showed a behaviour which was more similar to ProtElectra, with the learning rate being most important (30% and 36%, respectively). From this, we conclude that ProtXLNet finds it difficult to introduce the gene usage in the classification block, as it is trying to generate a better representation from the transformer block.

### 3.2 Comparing performances of transformer models

**Figure 2.**
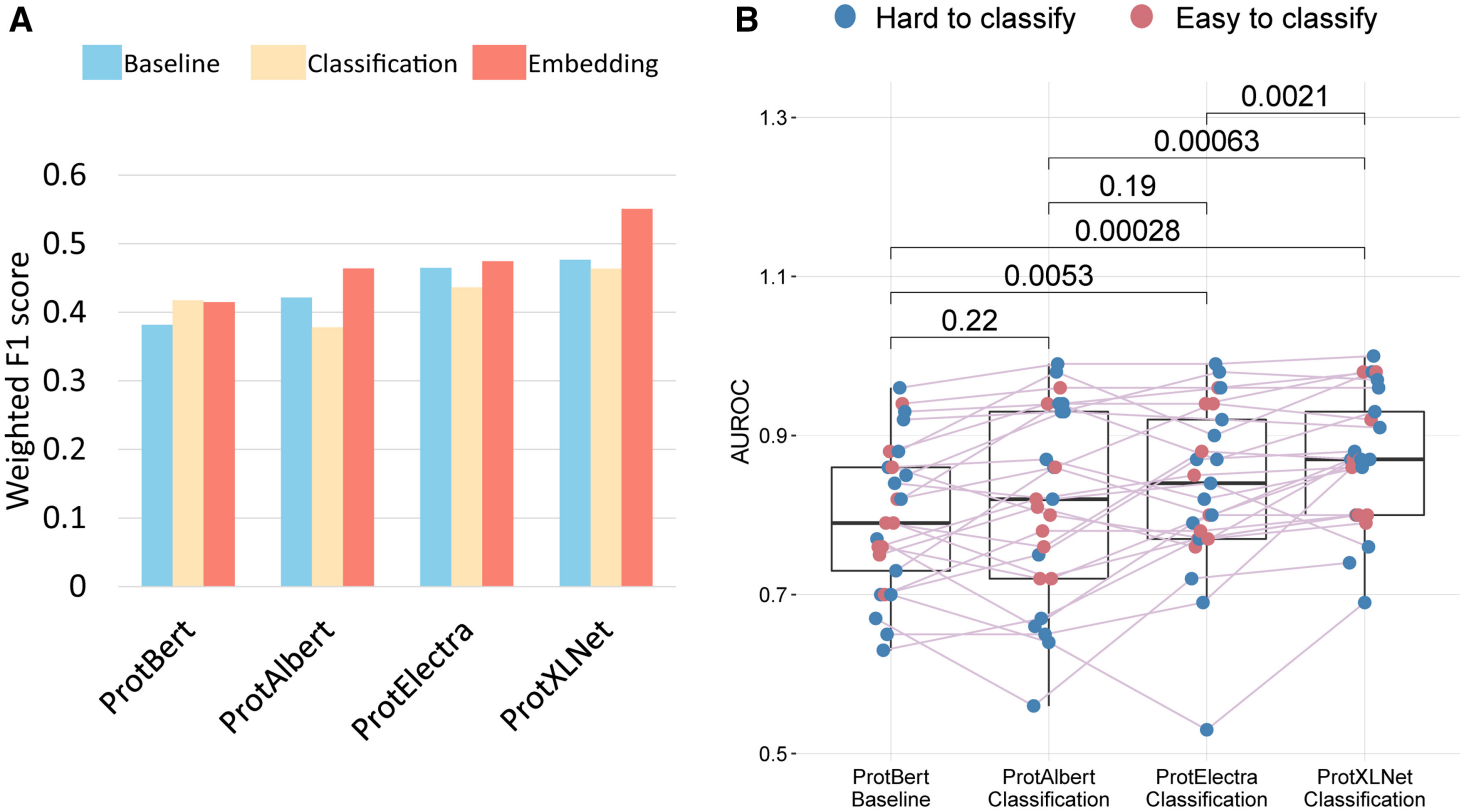
Comparison metrics for all transformer models. (A) Weighted F1-score on test dataset. (B) AUROC scores for the different classes in the test dataset. Only the significant *P* values as calculated by Wilcoxon paired test are mentioned in the plot.

When comparing the weighted F1 scores of the transformer models, we see a general trend in which ProtXLNet performed better than ProtElectra, while ProtElectra performed better than ProtAlbert. ProtBert performed the worst (Fig. 2A, Table 2, Supplementary Table S3). Moreover, we see another trend that the Embedding setting performed the best, followed by the Baseline setting. The Classification setting performed worst, although for ProtBert, the classification setting performed the best. On contrary, we observed that the Embedding setting outperformed the other settings for the rest of the transformer models, i.e. ProtAlbert, ProtElectra, and ProtXLNet. In this setting, the number of unclassified and misclassified labels reduced significantly (Supplementary Fig. S3).

When comparing the mean AUROC scores of all classes within the TCR–epitope dataset across the best (F1 score) setting for each of the four transformer models, we observed that the AUROC of the Classification setting of ProtBERT improved the performance over ProtAlbert in the Embedding setting. The Embedding setting of the ProtXLNet consistently performed significantly better than the other methods (Wilcoxon paired comparison, all three *P*-value’s <0.0021) and has the least unclassified and misclassified labels (Supplementary Fig. S4).

Twenty-five classes in the dataset were divided into ‘easy-to-classify’ classes and ‘hard-to-classify’ classes (Section 2). In line with the label classification, we observed that some that some epitopes are hard to classify by all the models, e.g. the CMV and EBV epitopes (Supplementary Fig. S4). Some models have problems to recognize specific classes, e.g. the Baseline setting of ProtBert cannot identify seven ‘hard-to-classify’ category (Supplementary Fig. S4) most likely due to very low number of training samples for these classes. When investigating the AUROC scores for the different categories of classes (Fig. 2B), we observed that there is hardly any difference between the AUROC scores for the ‘easy-to-classify’ classes and ‘hard-to-classify’ classes for ProtAlbert, ProtElectra, and ProtXLNet (scores are evenly distributed) (Supplementary Table S3). ProtBERT, however, seemed to struggle more with some of the ‘easy-to-classify’ classes. When studying the differences in classification accuracy between the ‘easy-to-classify’ classes and ‘hard-to-classify’ classes for the different settings for each transformer, we found no significant differences (Supplementary Fig. S5). Although based on the performance of the models, we observed that the Classification setting is the most economical as it used the least training time when compared with other methods of the models (Table 2).

### 3.3 Transformers in comparison with publicly available tools

To assess the best-performing transformer model from our study, we selected a few previously available best-performing tools classifying TCR–epitope pairs: TCRGP, TCRdist, and DeepTCR. As all the tools used a common database (VDJdb) for assessing the performances, a head-to-head comparison was possible. However, not all the epitopes were present in all the tools, since the database updates the samples in the classes. TCRGP and TCRdist lacked 1 epitope, whereas with the DeepTCR only 20 epitopes were in common to the epitopes used in our study. These classification tools were compared to our best model; ProtXLNet in the Embedding setting.

**Figure 3.**
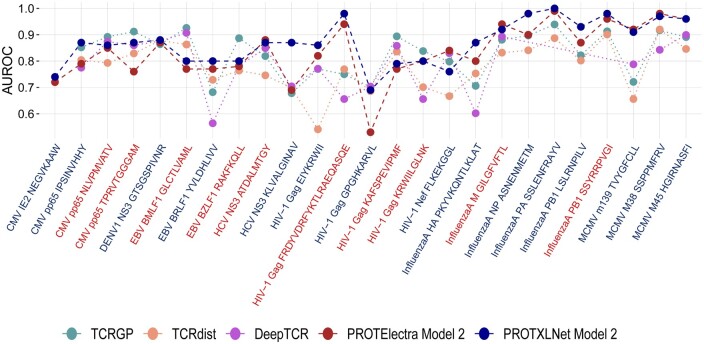
AUROC scores for the ProtXLNet model with embedding setting and previously known methods (TCRGP, TCRdist, and DeepTCR) for different epitope classes. The classes are coloured blue if the epitope is considered to be of the hard-to-classify class and red for the easy-to-classify class.

Our ProtXLNet model, which had a mean AUROC of 0.876 across the 24 common epitopes, showed an improvement of almost 5% when compared with TCRGP (AUROC = 0.831) and TCRdist (AUROC = 0.781) (Fig. 3). For the three ‘hard-to-classify’ labels of the MCMV epitope family, TCRGP and TCRdist had a mean AUROC of 0.843 and 0.807, respectively, whereas ProtXLNet had a mean AUROC of 0.946 for the same labels of MCMV epitope family (an improvement of ≥10%) (Supplementary Table S4).

Similarly, for the 20 common epitopes with DeepTCR model, ProtXLNet exhibited a mean AUROC of 0.856, whereas DeepTCR had a mean AUC of 0.784. This difference in the mean AUROC was mainly attributed to the ‘hard-to-classify’ labels, for which DeepTCR performed poorly (Fig. 3, Supplementary Table S4).

### 3.4 Benchmarking performance of our method

We used the IMMREP benchmark dataset and performed the experiments using our models for the four shared epitopes (CMV pp65 NLVPMVATV, CMV pp65 TPRVTGGGAM, EBV BMLF1 GLCTLVAML, and InfluenzaA M GILGFVFTL). We observed that for these epitopes both for IMMREP dataset and our dataset ProtXLNet Embedding setting out-performed all the models (Table 3). Moreover, we observed consistently comparable AUC for all the models in both the datasets for these epitopes.

**Table 3.** Assessing the performance of all the protein-BERT models trained in our study for four common epitopes in IMMREP benchmark dataset.

		IMMREP dataset				Our dataset			
		CMV pp65 NLVPMVATV	CMV pp65 TPRVTGGGAM	EBV BMLF1 GLCTLVAML	InfluenzaA M GILGFVFTL	CMV pp65 NLVPMVATV	CMV pp65 TPRVTGGGAM	EBV BMLF1 GLCTLVAML	InfluenzaA M GILGFVFTL
ProtBERT	Baseline	0.69	0.81	0.83	0.87	0.75	0.81	0.86	0.81
	Classification	0.8	0.86	0.89	0.87	0.76	0.75	0.76	0.82
	Embedding	0.61	0.54	0.77	0.84	0.79	0.74	0.63	0.76
ProtAlbert	Baseline	0.81	0.87	0.9	0.89	0.85	0.70	0.70	0.84
	Classification	0.78	0.79	0.91	0.87	0.77	0.73	0.72	0.83
	Embedding	0.83	0.93	0.93	0.9	0.82	0.81	0.72	0.86
ProtElectra	Baseline	0.84	0.86	0.86	0.91	0.88	0.78	0.77	0.92
	Classification	0.8	0.88	0.92	0.89	0.71	0.79	0.75	0.85
	Embedding	0.84	0.89	0.94	0.91	0.85	0.76	0.77	0.94
ProtXLNet	Baseline	0.85	0.91	0.93	0.91	0.85	0.80	0.71	0.89
	Classification	0.81	0.94	0.91	0.9	0.82	0.79	0.80	0.86
	Embedding^ϕ^	0.89	0.98	0.96	0.93	0.86	0.87	0.80	0.92

ϕ The ProtXLNet Embedding setting out-performed other models for IMMREP and our dataset.

Additionally, we used the performance metrics available for the common epitopes by the sequence-similarity-based model (Baseline-SS) method in the suggested article (Montemurro *et al.* 2021). Only 4 labels (i.e. CMV pp65 NLVPMVATV, EBV BMLF1 GLCTLVAML, HCV NS3 ATDALMTGY, and InfluenzaA M GILGFVFTL) out of 25 labels were overlapping. When comparing the AUC for these four labels, we found that the two models achieved comparable performance with a minor advantage of ProtElectra Embedding setting when tested on the task of predicting the positive versus 10× negatives (Table 4). However, ProtElectra Embedding setting significantly outperformed the Baseline-SS for all evaluations when separating between positives and swapped negatives as also mentioned by the NetTCR prediction performances (Montemurro *et al.* 2021).

**Table 4.** Comparing performance of ProtElectra Embedding setting, best performer in our study, to the Baseline-SS model based on TCR sequence-similarity.

	ProtElectra Embedding setting	Baseline-SS	
		Baseline-SS Pos versus True Neg	Baseline-SS Pos versus Swap Neg
CMV pp65 NLVPMVATV	0.85	0.75	0.72
EBV BMLF1 GLCTLVAML	0.77	0.84	0.77
HCV NS3 ATDALMTGY	0.78	0.84	0.76
InfluenzaA M GILGFVFTL	0.94	0.85	0.77

Finally, we assessed the performance of our best model, ProtXLNet Embedding setting, by randomly shuffling the labels of our validation dataset. We observed that the AUROC of randomly labelled validation data is 0.49 suggesting that performance of our model is not predicted by chance (Supplementary Table S5). Furthermore, we constructed a neural network model (NN) using TensorFlow and Keras, comprising an input layer for *V* and *J* features, two hidden layers with ReLU activation functions, and an output layer with softmax activation. The model was trained using categorical cross-entropy and evaluated on the test set. Although the simple NN model with *VJ* genes as input had a good performance our best-performing model demonstrated 22% improvement in compared to *VJ* genes NN model (Supplementary Table S6).

## 4 Discussion

A diverse set of TCRs is generated by the adaptive immune system that recognizes a short immunogenic peptide on the proteins of the pathogens. The epitopes should ideally have an explicit set of TCRs with particular properties, including distinct CDR3 sequences, gene usage, and MHC class. Several sequence-based and deep learning-based models have been proposed to predict TCR–epitope pairs; however, there is still a performance deficit in these methods. In this study, we used a novel deep learning approach, transformers, which uses attention layers that are learnt to focus on relevant parts of the provided TCR sequence. We adopted three pre-trained auto-encoders (ProtBERT, ProtAlbert, and ProtElectra) and one auto-regressor (ProtXLNet) and added a classification head to build multi-class classification models to identify TCR specificity to the selected epitopes from the VDJdb. To incorporate gene usage in these transformers, we have made novel modifications to these transformers, either by adapting the classification block (Classification setting) or by adapting the embedding layer (Embedding setting).

Across 12 different transformer models, we found that the ProtXLNet auto-regressor model, when modified with the Embedding setting, achieved the highest weighted F1 score (0.55), the highest Mean AUROC for the ‘hard-to-classify’ samples (0.88) as well as for the ‘Easy-to-classify’ samples (0.86). Additionally, it demonstrated significantly better AUC values for all classes, which are not predicted by chance and are better than *V/J* identities alone (Supplementary Table S3). Moreover, this best-performing transformer model outperforms previously developed sequence-based (TCRGP and TCRdist) and deep learning (DeepTCR)-based methods (Supplementary Table S4).

We also assessed the performance of our trained model in the benchmark IMMREP datatset, where ProtXLNet Embedding setting performed the best for the four common labels in IMMREP dataset. Similarly, when comparing with the baseline-SS model, ProtElectra Embedding setting significantly outperformed the Baseline-SS model for all evaluations when separating between positives and swapped negatives, similar to the NetTCR performance (Montemurro *et al.* 2021).

The results can further be evaluated using attention head visualization in which attention heads playing the most vital role towards a classification can be highlighted. As we can instruct the tokenizer to calculate attention for specific regions in CDR3 sequences, identifying the relevant amino-acid residues from the interaction of TCR and epitope by the attention mechanism can further improve the usage of the transformers for making better predictions of specific TCRs (at the level of amino-acids in the CDR3 sequences) binding to specific epitopes.

The previous methods have shown that incorporating CDR3 sequences and gene usage information of both alpha and beta chains along with MHC restriction can increase the performance and the prediction power of the tools. Here, in our study, we only used the CDR3 sequences and gene usage of the TCR beta chain. We only targeted this simplified approach because our main goal was to understand the different ways to incorporate gene usage within the transformer models (i.e. the Classification setting and the Embedding setting).

During optimization of the different parameters for all four transformer models, we observed that the models tended to stop early for potentially good runs. This might have been caused by the choice of the loss function, as, depending on modulating factor, the focal loss may cause many spikes in loss values. These spiking fluctuations may then be misinterpreted and cause early stopping. Increasing the tolerance threshold for early stopping led to stale runs and immense waste of training time. Exploring how to best tackle this problem could increase the optimization.

A common theme for developing tools to predict TCR specificity to the epitopes is to improve encoding of input data, e.g. as observed in the tools developed using sequence similarity (Pogorelyy *et al.* 2019), k-mer sequence features (Tong *et al.* 2020), convolutional models (Montemurro *et al.* 2021), utilizing amino acid physicochemical properties (Gielis *et al.* 2019), learning a non-parametric function (Jokinen *et al.* 2021) or enhancing sequence feature into a high-dimensional space (Sidhom *et al.* 2021), utilizing molecular structures (Weber *et al.* 2021), or pre-training BERT model on TCR data (Wu *et al.* 2021). Generally, these tools train a binary classification model to exhibit epitope specificity among a background of epitopes. However, our study provided an alternative approach: a multi-class classification model towards TCR classification. Only DeepTCR included a multi-class setting, but this setting was employed exclusively for the GAG TW10 epitope family and not for the entire TCR data (with 10 variants). To the best of our knowledge, the multi-class approach has never been utilized in such a fashion because of scarcity of TCR data or lacking computational resources. We could overcome these limitations by utilizing pre-trained transformers, trained on large corpus of protein sequence data.

We showed that the ProtXLNet transformer with the Embedding setting outperformed the previously known sequence-based and deep learning-based algorithms. However, a recently published transformer-based algorithm, i.e. TCR-BERT, was not included. TCR-BERT used two different BERT models (one for beta sequences and one for alpha sequences) and combined their embeddings for the classifier block. It does not exploit additional information, like we do on the gene usage. Despite not being able to make a head-to-head comparison with TCR-BERT, we assessed the comparisons based on the AUROCs reported by the TCR-BERT method, but that could only be done for the CMV-pp65-NLVPMVATV class. The TCR-BERT approach resulted in an AUROC of 0.837 (using both TRA and TRB sequences), whereas our modified ProtXLNet model had an AUROC of 0.86 (using only TRB sequences). TCR-BERT utilized the entire 81K entries from VDJdb to pre-train a BERT model as opposed to us. We used pre-trained transformer models built from proteins in either Uniref or BFD, comprising 216 million and 2.122 billion protein sequences. For fine-tuning the classification head, we utilized only the VDJdb data with a confidence score of more than 0. TCR-BERT fine-tuned TCR specificity for binding to a positive class (epitope). Nevertheless, a more in-depth comparison of the two approaches could provide greater insight into which approach (fine-tuning or pre-training) is better suited for TCR data.

Although our approach demonstrates the potential of transformers by fine-tuning CDR3 sequences and how additional features can be juxtaposed cohesively, some limitations remain. Problematic events such as hallucination and catastrophic forgetting (Sun *et al.* 2019) were not evaluated. These problems are prevalent in deep neural networks and contribute significantly to misclassification. Hallucination occurs in generated sequence at the last transformer block, leading to unlikely content. Predominantly, hallucination (Kolouri *et al.* 2019, Ji *et al.* 2022) often occurs when generating natural languages. As we just explored classification through transformers and not generation of sequences, we do not expect to suffer from hallucination. Catastrophic forgetting is prevalent in transformers where the weights learned during pre-training get overridden during fine-tuning. Therefore, we employed early stopping and we lowered the learning rate to prevent pre-trained knowledge from being overridden (or diminished) during fine-tuning. Furthermore, the fine-tuning and henceforth the performance of all the BERT models tested with Baseline setting could be further improved by freezing technique model blocks in BERT models (Liu *et al.* 2021); however, its feasibility with the modified BERT model is still limited.

Taken together, we have shown the potential of transformers and multi-class learning for TCR–epitope pair prediction. The comparison with pre-existing prediction methods for TCR–epitope pairs is performed by comparing the AUROC presented in their studies which could be a limiting factor as the underlying data can have a little variation. Although transformers outperformed other methods, fine-tuning of the hyperparameters can further enhance the performance of transformers.

## Supplementary Material

btad632_Supplementary_Data

## Data Availability

The data used is cited and no data was generated in our research.
