# Supplementary material for: Determining epitope specificity of T-cell receptors with transformers
Source: Bioinformatics. 2023 Oct 17;39(11):btad632. doi: 10.1093/bioinformatics/btad632 (PMC10636277; doi:10.1093/bioinformatics/btad632)
Supplement: btad632_Supplementary_Data [file btad632_supplementary_data.docx]

Determining epitope specificity of T-cell receptors with Transformers

Abdul Rehman Khan^1^, Marcel JT Reinders^1,2^ and Indu Khatri*^2,3^

^1^Pattern Recognition and Bioinformatics, Delft University of Technology

^2^Leiden Computational Biology Center, Leiden University of Medical Center.

^3^Department of Immunology, Leiden University of Medical Center.

*To whom correspondence should be addressed.

# Indu Khatri Email: [indu2287@gmail.com](mailto:indu2287@gmail.com); [i.khatri@lumc.nl](mailto:i.khatri@lumc.nl)

Supplementary Text

*Biology behind antigen recognition by T-Cell*

Understanding T cell receptors (TCRs) relates directly to the understanding of mechanisms involved in the adaptive immune system. While adaptive immunity involves both B and T cells, we will focus on T cells which will provide us insight into cell-mediated adaptive immunity.

### Antigen Presentation and MHC Restriction

Presenting peptides to the TCRs is done through a class of cells known as antigen-presenting cells (APC) and is presented through Major Histocompatibility complexes (MHC); this process is termed antigen presentation. Degradation of antigen is done inside APC; this degradation is undertaken through two different pathways depending on if MHCI or MHCII is expressed on the APC. The affinity of degraded peptide (or epitope) to the MHC dictates which peptide will be presented by the MHC and also to which T-Cell (CD4+ or CD8+) (Zareie, Farenc, & La Gruta, 2020).


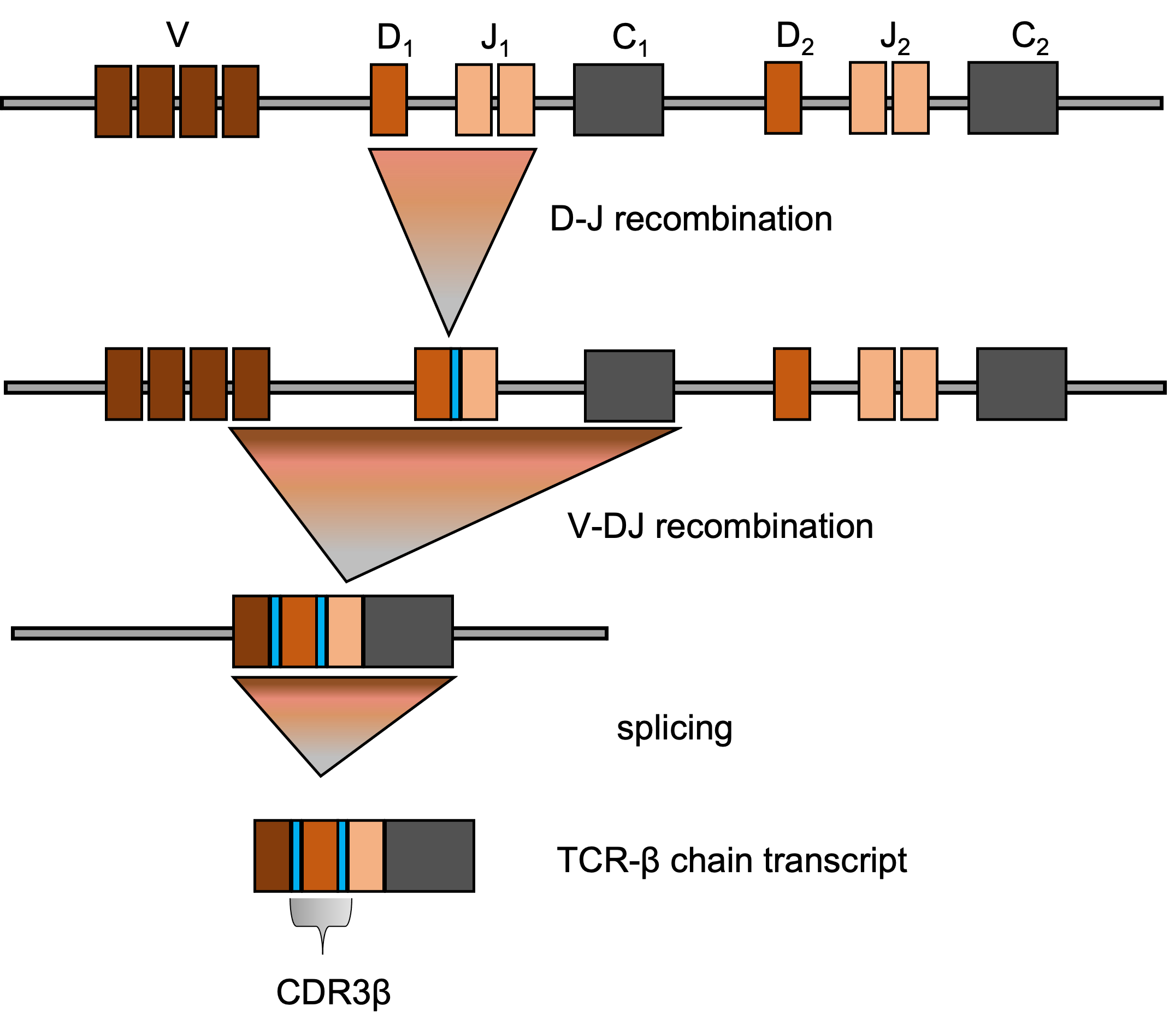


**Figure 1: TCRβ gene rearrangement and structure.** Overview of V(D)J recombination. Region in blue indicates junctional sites which are assembled by random additional and deletion. CDR3β region shown in grey on TCR protein.

### Antigen Recognition

TCR expressed on either CD8+ or CD4+ T cells binds to MHC for antigen recognition by their respective T cells. T cells' clonal nature dictates the unique binding site on its TCRs and hence its specificity. T-Cell consists of Variable (V) and Constant (C) region; it is on V region where the sequence diversity is the most concentrated and is present on both Alpha and Beta chain of TCRs. TCR genes undergo *V*, *D* and *J* gene rearrangement providing TCR with high diversity.

### Gene rearrangement

Although Gene rearrangement provides TCR diversity expected at 10^18^ in humans and 10^15^ in mice, it is not random at all, and thus regulation is governed by multiple factors (Attaf, Huseby, & Sewell, 2015) (**Figure 1**). TCR beta locus comprises 46 *V* gene segments, followed by two groups of *D*, *J* and *C* gene segments (*D1*, *J1*, *C1* or *D2*, *J2*, *C2*). *D* to *J* recombination first occurs between a *D* gene and one of *J* gene of first group or *D* and *J* gene of the second group; followed by *V* to the newly rearranged *D* and *J* gene. *V(D)J* recombination of TCR genes plays a vital role in governing the diversity of a repertoire (all unique TCR within an individual’s immune system). Additionally, random insertion and deletion of nucleotides at junctions of *V*, *D* and *J* give rise to hypervariable regions, known as complementarity determining regions.

### Complementarity-determining region on TCR

Complementarity-determining regions are present at the junction of V and D segment of TCRs Fig.8. This region of TCR interacts the most with both MHC and epitope for recognition, which is due to its hypervariability. Two types of TCR, i.e., TCRAB and rarely TCRGD are mounted by the adaptive immune system for an immune response. These receptors (at protein level) are generated from two different chains, i.e., TCRAB from TCRA and TCRB and TCRGD from TCRG and TCRD. Each of these four receptors is generated from the recombination of *V*, *D* and *J* genes from their individual loci. Several *V*, *D* and *J* genes for TCRB and TCRD and *V* and *J* genes for TCRA and TCRG are distributed over a long stretch of chromosomes 7 and 14 in the human genome. Any of these *V*, *D* and *J* genes can be selected for each locus and any of the chains, i.e., TCRA/TCRB can be randomly selected to generate diverse receptors to raise an efficient immune response against infections. Overall, 10^16^ receptors can alone be generated by the *V(D)J* recombination events. Apart from *V(D)J* recombination, another factor that adds to the diversity of these receptors is the addition of nucleotides on both sides of D gene during *V(D)J* recombination. This region is known as complementarity determining region 3 (CDR3), which is fundamental in interacting with and recognising the antigen. Determining binding specificity to an antigen thus helps us to assess the immune system's ability to engage with pathogens and also to evaluate the response undertaken by it.

*Transformers*

Transformers are a type of Sequence to Sequence (or Seq2Seq) model that transforms a source sequence's representation to a representation of a target sequence. Machine translation (also known as sequence transduction) which were widely performed using LSTM (long-short-term-memory) models or RNN (recurrent neural networks) models, had limitations. Limitations of fixed sequence length in traditional methods (and even the bottleneck between encoder and decoder architectures) were then overcome with the introduction of Transformers. Transformers retained the encoder-decoder architecture and introduced a new form of attention mechanism, self-attention, which outperformed standard practices.

### Encoder-Decoder Architecture

Given a source sequence to be translated to a target sequence, the encoder would generate vector representation for each word in the source sequence, and the decoder would then read these vectors and generate words in the target sequence. Certain limitations were discovered; translating word to word doesn't account for languages written from left to right or with different grammar compositions. Subsequently, to reduce computational resources instead of encoding the entire source sequence, a need to focus on relevant words arise.

Recurrent Neural Network (Bahdanau, Cho, & Bengio, 2014) would solve the former issue by including a weighted sum of preceding and succeeding words in a sequence whilst encoding a word. It employed RNN to encode each word, and a decoder would then decode from this representation. With the introduction of LSTMs (Sutskever, Vinyals, & Le, 2014) long-range dependencies for a word were also accounted for.

Ditching recurrent and convolutional layers was Transformers, replacing it with an attention mechanism. Transformers introduced encoding through self-attention, which would amplify the contribution of relevant words and diminish the contributions of irrelevant words while encoding a vector representation for a word. All this whilst retaining encoder-decoder architecture (there are encoder-only architectures like BERT).

### Attention, Self-attention and multi-head attention

Origins of attention lie in the field of psychology, where the observations in behavioural patterns were attributed to where the brain was paying attention. The brain preserves computation resources by paying attention to only crucial details to reach an answer (Lindsay, 2020), which can be mimicked by utilising a weighted sum of relevant words while encoding a word. Mathematically formulating this "flexibility" in a neural network is known as the attention mechanism. Global and Local attention are some of the early attention mechanisms which were applied in computer vision (Luong, Pham, & Manning, 2015; Xu et al., 2015). With the introduction of transformers, self-attention came into the limelight.

While traditional attention mechanisms would include the contribution of surrounding words blindingly, self-attention would also include the position of the word to introduce the sense of context in encoding a word. In summary, similar words in different positions in a sequence would get different encoding.

Fig. 9 shows encoding the second word in the output sequence. Three different representation of input is used to calculate self-attention. Learning the weights for every three representations is equivalent to learning self-attention. Query and Key are used to compute scores, which undergoes some processing before taking a dot product with the Value representation. This dot product will enhance values in the Value matrix, which corresponds to higher relevance and hence augment the effect of those words into the resulting representation of the word. This can be done multiple times in parallel for a single word, termed as multi-headed attention. Multi-headed attentions allow transformers to accommodate relevance from multiple positions in a sequence.

### Transfer Learning and Transformers architectures

Training a transformer involves the concept of transfer learning; in transfer learning, we divide training a model into two different parts: pre-training and fine-tuning. Pre-training involves two tasks mask language modelling and Next Sentence prediction. In the case of ProtTrans, only mask language modelling was utilised. The weights learned in pre-training are fine-tuned on downstream tasks, consequently saving time and resources in training a transformer from the beginning and prime advantage of transfer learning. Additionally, different Transfer models employ different approaches for pre-training tasks, which is motivated by their methodology.

There are two architectures utilised in this work, Auto-encoder and Auto-regressive. BERT, Albert, Electra are auto-encoder models with only encoders and no decoders. XLNet is an autoregressive language model. While BERT learns bidirectional language modelling, ALBERT (A lite BERT) is a more efficient version of BERT with parameter sharing among different encoder layers. Both of them use the same mask language modelling. On the other hand, ELECTRA utilises Generator-Discriminator based approach; the discriminator then detects corrupted tokens generated by the generator during masked language modelling. The discriminator trained is then used for fine-tuning on downstream tasks.

While auto-encoder models reconstruct original data during mask language modelling. They lack obvious information; masked token (it is a type of special token signified as MASK by tokeniser) will not be seen in downstream tasks, dependency learnt for the masked token (and the original token) is then not transferred (or would never be needed); termed as the pretrain-finetune discrepancy. The pretrain-finetune discrepancy is addressed in XLNet. XLNet is an auto-regressive model which implements two-stream self-attention as a means to address both forward and backward dependencies as well as to address pretrain-finetune discrepancy. Providing different factorisation orders during permutation language modelling enables the model to gather positional information from a possible position for a given token.

On the other hand, two-Stream self-attention uses additional self-attention to isolate the content while the model learns positional information (or context) during pre-training (**Figure 2**). Any difference between XLNet and BERT is due to this very difference in pre-training objective, which helps it retain a more significant number of dependencies than BERT. In summary, XLNet is a bidirectional transformer similar to BERT but utilises permutation language modelling.


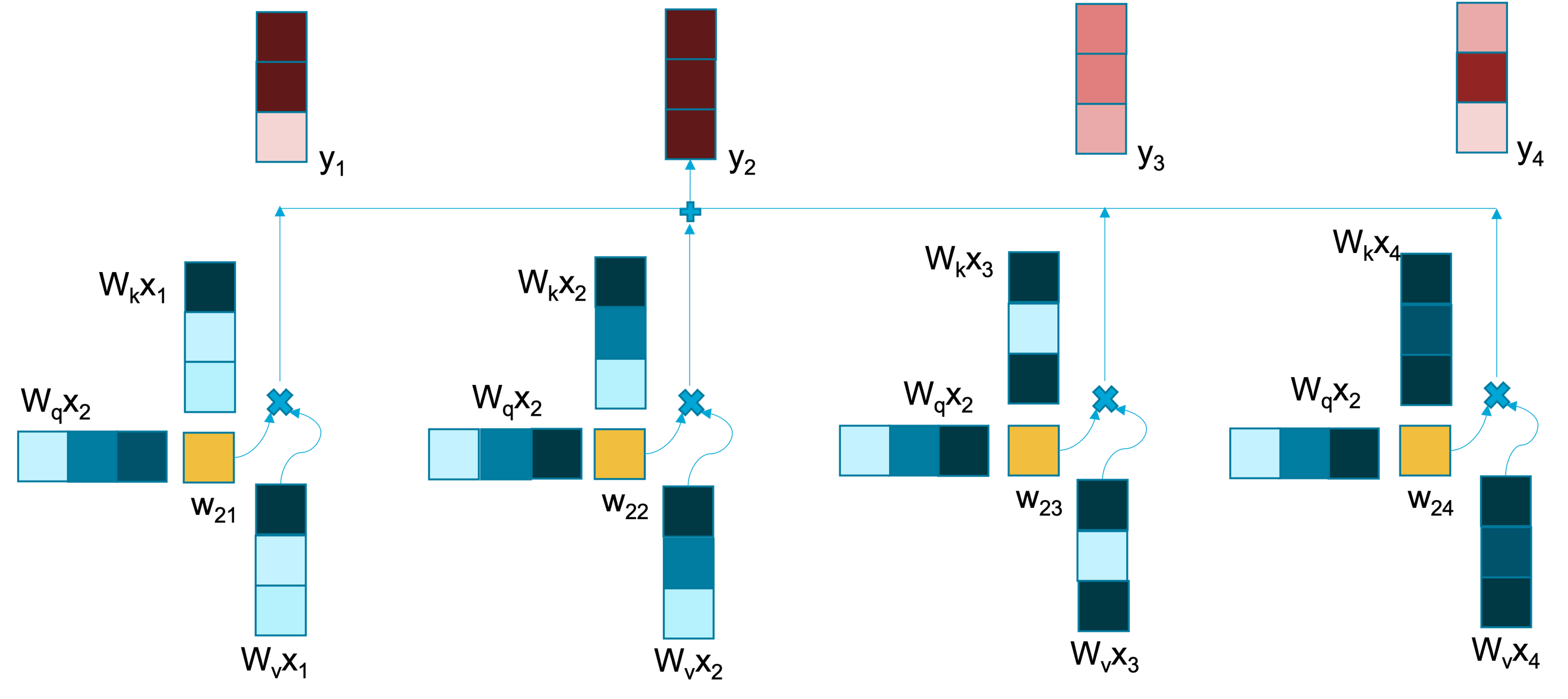


**Figure 2: Self-Attention in Transformers.** Overview of self-attention computation for encoding y_2_. Two representations (Query and Key) of a word are used to compute scores (shown in yellow) which enhances or reduces effect of a word (multiplying by Value). Resultant effect is summed across all words to encode a single word (y_2_)

**Supplementary Figures and Tables**

**Supplementary Figure 1**

**Supplementary Figure 1: 25 Epitope labels used as classes for the classification of TCR to epitopes after filtering steps as mentioned in Table 1.** The classes are colored blue if the epitope is considered as hard to classify (<100 samples) and red for easy to classify labels (>100 samples).

**Supplementary Figure 2**

**Supplementary Figure 2: Performance of the hyperparameters for all considered transformer models.** The bar graphs are colored based on the methods implemented on the different transformer models.

**Supplementary Figure 3**

**
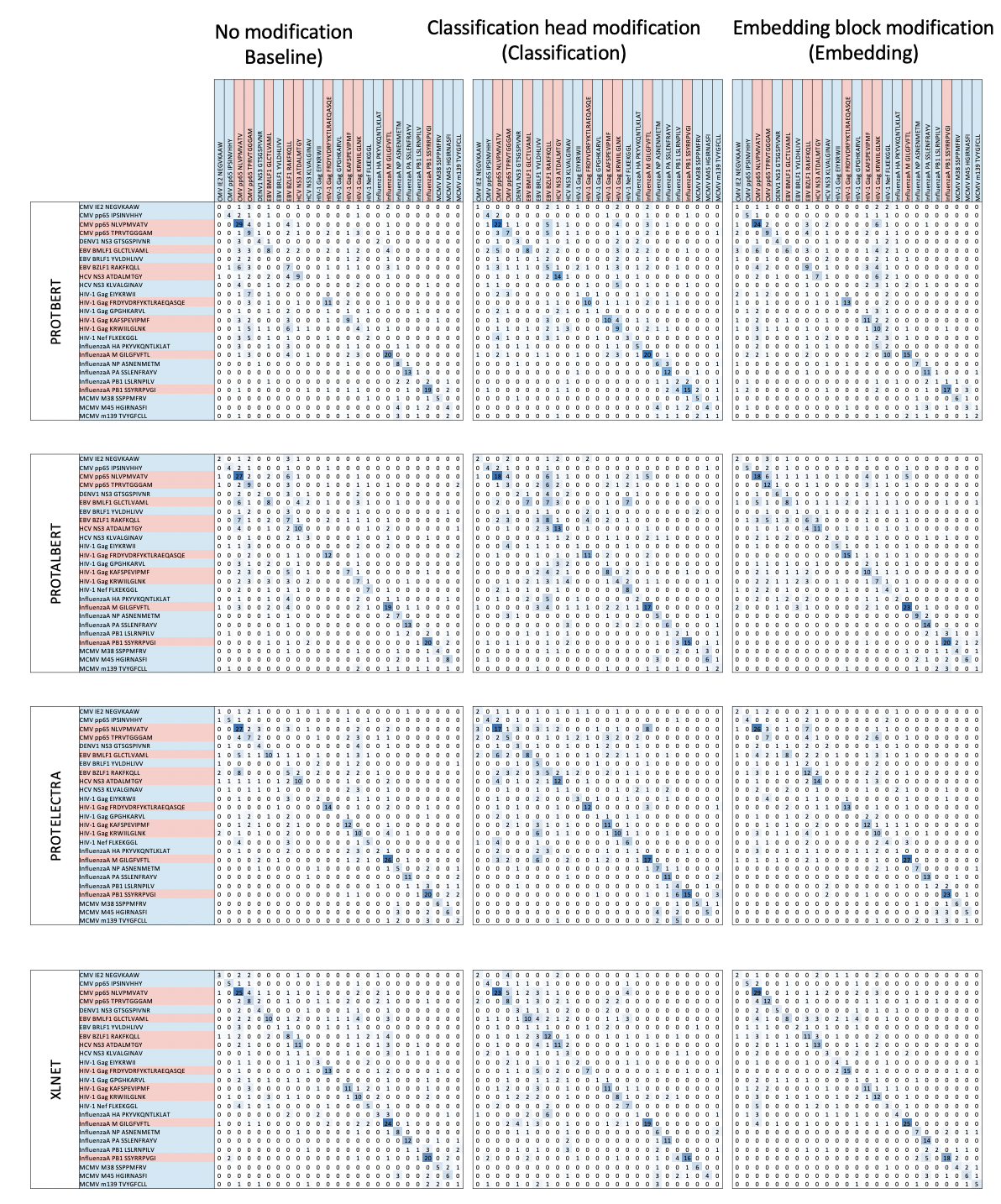
**

**Supplementary Figure 3: Confusion matrix of the performance of the four optimized transformers for all the three methods.** The labels are colored based on their classification i.e. hard (blue) or easy (red) to classify. The matrix itself is colored from low (white) to high (blue) correctly classified labels.

**Supplementary Figure 4**

**Supplementary Figure 4: The comparison of the AUC values of the different transformer models for all the three methods A) ProtBert; B) ProtAlbert C) ProtElectra; and D) ProtXLNet.** The points are colored based on the classification of the epitope classes i.e. hard (blue) or easy (red) to classify. The p values are indicated for the individual comparisons.

**Supplementary Figure 5**

**
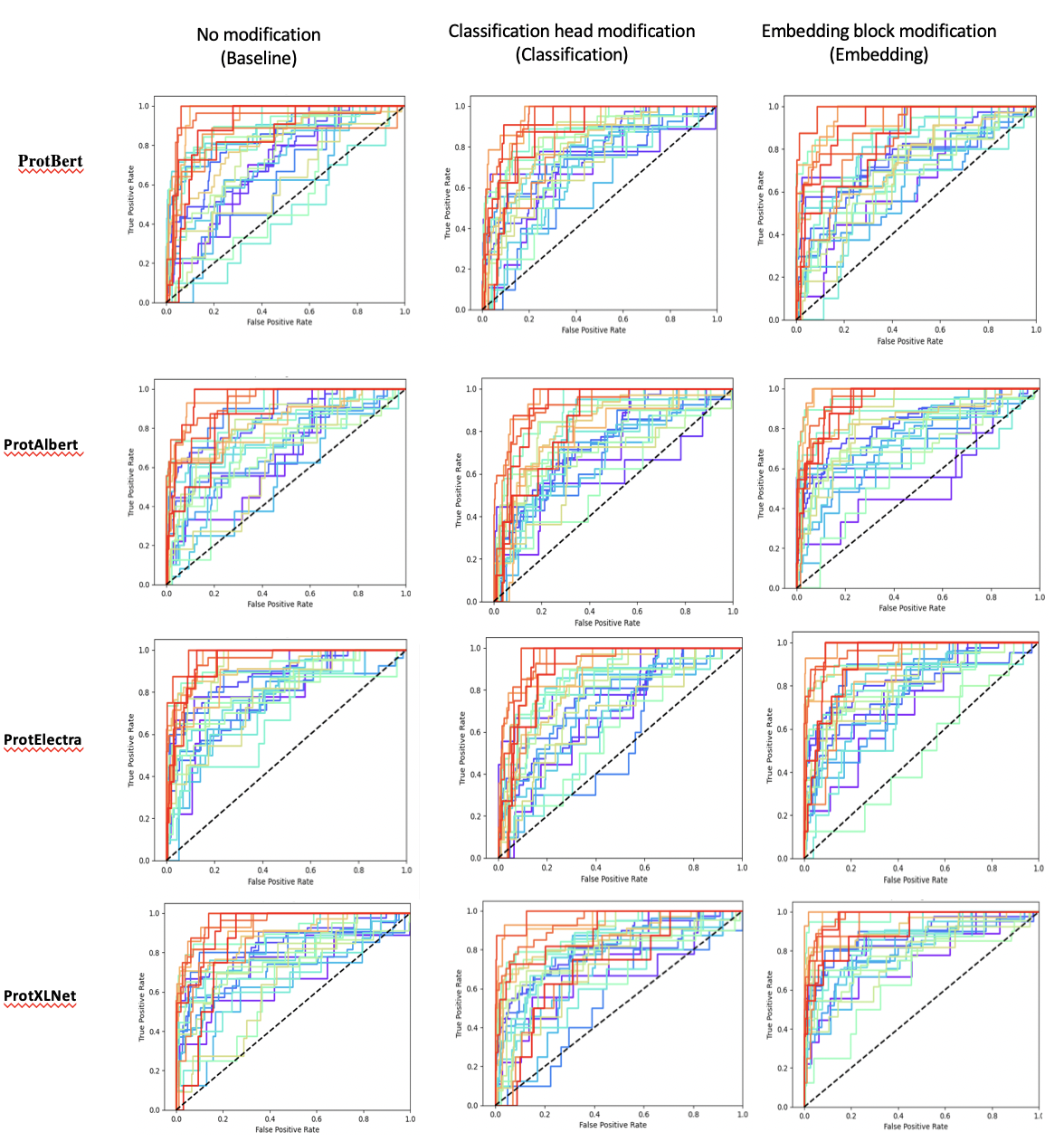
**

**Supplementary Figure 5: ROC plots of the performance of the 25 classes for four transformers for all the three methods.** The labels are highlighted based on their classification i.e. hard (blue) or easy (red) to classify.

**Supplementary Tables:**

**Supplementary Table 1: The list of tested hyperparameters with range of considered values (“modulations”).**

| **Hyperparameters** | **Modulations** |
| --- | --- |
| Gamma | (0, 10) with step of 0.5 |
| Adam beta 1 | [0.5, 0.9] with step of 0.01 |
| Adam beta 2 | [0.5, 0.99] step of 0.001 |
| Learning rate | [10-5, 10-2] from log domain |
| Weight decay | 0, 0.01, 0.001, 0.0001, 0.00001, 0.000001 |
| Gradient accumulation steps | [1, 128] |
| Classifier / Summary layer dropout | [0.5,0.9] with step of 0.1 |
| Hidden layer / dropout | [0.5, 0.9] with step of 0.1 |
| Attention layer dropout | [0.5, 0.9] with step of 0.1 |
| Warmup ratio | 0.10, 0.20 |
| Seed (for model stability) | [1, 100] |

**Supplementary Table 2: The 25 classes and corresponding count of TCRs.** Each class comprise of the organism (e.g. CMV, DENV), protein name (e.g. IE2, pp65), and epitope protein sequence (e.g. NEGVKAAW, IPSINVHHY). The classes are highlighted based on their classification i.e. hard (blue) or easy (red) to classify.

**
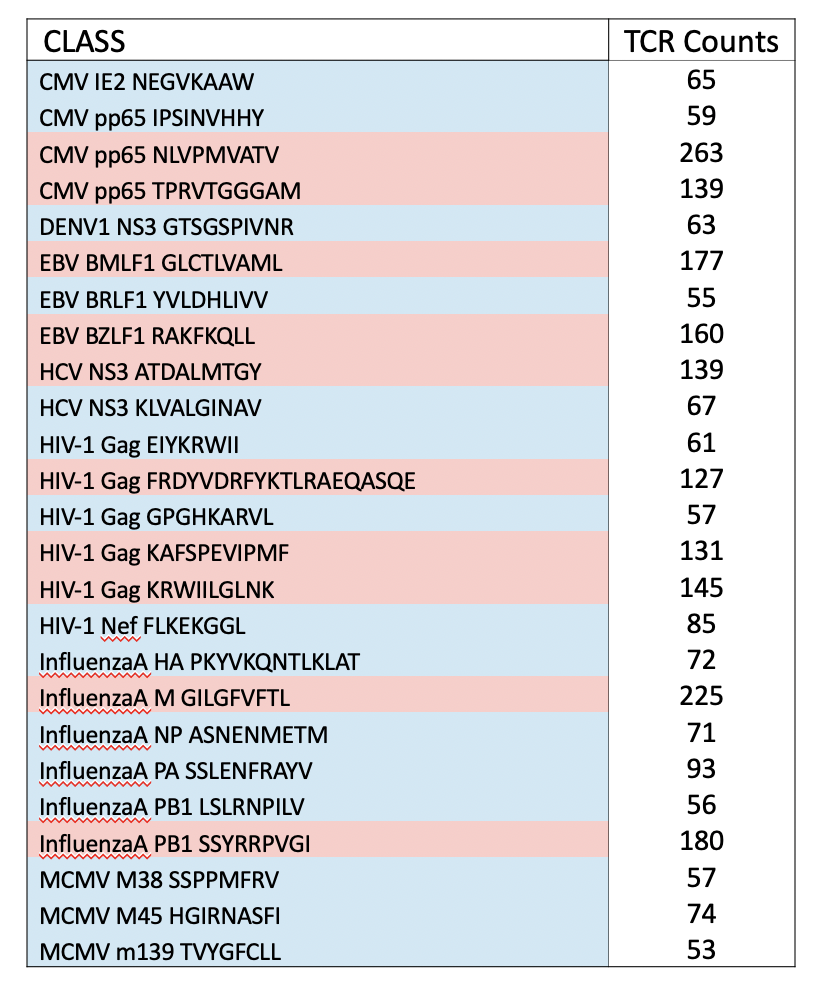
**

**Supplementary Table 3: The AUC values corresponding to the ROC plots in Supplementary Figure 6.** The classes are highlighted based on their classification i.e. hard (blue) or easy (red) to classify.

**
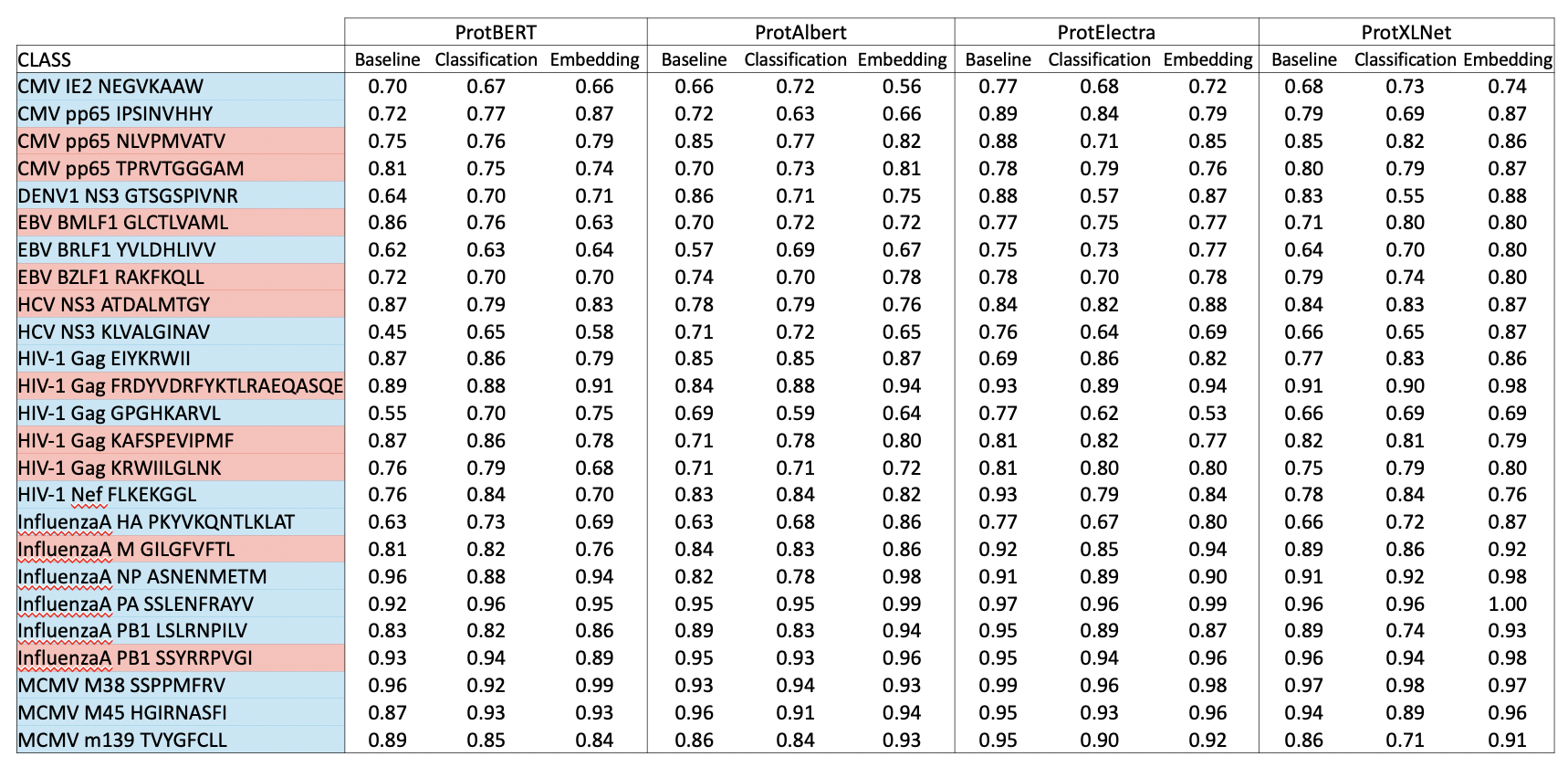
**

**Supplementary Table 4: The AUC values of the publicly available tools compared in the study**.

**Supplementary Table 5: The AUC values of the ProtXLNet Embedding setting with correct and randomly shuffled labels**

|  | ProtXLNet Embedding setting | |
| --- | --- | --- |
| CLASS | Correct labels | Randomly shuffled labels |
| CMV IE2 NEGVKAAW | 0.74 | 0.37 |
| CMV pp65 IPSINVHHY | 0.87 | 0.54 |
| CMV pp65 NLVPMVATV | 0.86 | 0.45 |
| CMV pp65 TPRVTGGGAM | 0.87 | 0.35 |
| DENV1 NS3 GTSGSPIVNR | 0.88 | 0.44 |
| EBV BMLF1 GLCTLVAML | 0.80 | 0.54 |
| EBV BRLF1 YVLDHLIVV | 0.80 | 0.60 |
| EBV BZLF1 RAKFKQLL | 0.80 | 0.52 |
| HCV NS3 ATDALMTGY | 0.87 | 0.41 |
| HCV NS3 KLVALGINAV | 0.87 | 0.46 |
| HIV-1 Gag EIYKRWII | 0.86 | 0.53 |
| HIV-1 Gag FRDYVDRFYKTLRAEQASQE | 0.98 | 0.34 |
| HIV-1 Gag GPGHKARVL | 0.69 | 0.53 |
| HIV-1 Gag KAFSPEVIPMF | 0.79 | 0.65 |
| HIV-1 Gag KRWIILGLNK | 0.80 | 0.51 |
| HIV-1 Nef FLKEKGGL | 0.76 | 0.66 |
| InfluenzaA HA PKYVKQNTLKLAT | 0.87 | 0.52 |
| InfluenzaA M GILGFVFTL | 0.92 | 0.55 |
| InfluenzaA NP ASNENMETM | 0.98 | 0.54 |
| InfluenzaA PA SSLENFRAYV | 1.00 | 0.49 |
| InfluenzaA PB1 LSLRNPILV | 0.93 | 0.34 |
| InfluenzaA PB1 SSYRRPVGI | 0.98 | 0.47 |
| MCMV M38 SSPPMFRV | 0.91 | 0.57 |
| MCMV M45 HGIRNASFI | 0.97 | 0.46 |
| MCMV m139 TVYGFCLL | 0.96 | 0.50 |

**Supplementary Table 6: The AUC values of the ProtXLNet Embedding setting and simple neutral network model trained using only VJ genes as input.**

| CLASS | ProtXLNet Embedding setting | Simple NN with VJ genes as input |
| --- | --- | --- |
| CMV IE2 NEGVKAAW | 0.74 | 0.53 |
| CMV pp65 IPSINVHHY | 0.87 | 0.67 |
| CMV pp65 NLVPMVATV | 0.86 | 0.71 |
| CMV pp65 TPRVTGGGAM | 0.87 | 0.74 |
| DENV1 NS3 GTSGSPIVNR | 0.88 | 0.87 |
| EBV BMLF1 GLCTLVAML | 0.8 | 0.66 |
| EBV BRLF1 YVLDHLIVV | 0.8 | 0.68 |
| EBV BZLF1 RAKFKQLL | 0.8 | 0.66 |
| HCV NS3 ATDALMTGY | 0.87 | 0.73 |
| HCV NS3 KLVALGINAV | 0.87 | 0.54 |
| HIV-1 Gag EIYKRWII | 0.86 | 0.56 |
| HIV-1 Gag FRDYVDRFYKTLRAEQASQE | 0.98 | 0.91 |
| HIV-1 Gag GPGHKARVL | 0.69 | 0.68 |
| HIV-1 Gag KAFSPEVIPMF | 0.79 | 0.79 |
| HIV-1 Gag KRWIILGLNK | 0.8 | 0.63 |
| HIV-1 Nef FLKEKGGL | 0.76 | 0.55 |
| InfluenzaA HA PKYVKQNTLKLAT | 0.87 | 0.7 |
| InfluenzaA M GILGFVFTL | 0.92 | 0.68 |
| InfluenzaA NP ASNENMETM | 0.98 | 0.78 |
| InfluenzaA PA SSLENFRAYV | 1 | 0.66 |
| InfluenzaA PB1 LSLRNPILV | 0.93 | 0.77 |
| InfluenzaA PB1 SSYRRPVGI | 0.98 | 0.81 |
| MCMV M38 SSPPMFRV | 0.91 | 0.82 |
| MCMV M45 HGIRNASFI | 0.97 | 0.86 |
| MCMV m139 TVYGFCLL | 0.96 | 0.79 |
